# The Ciliate *Paramecium* Shows Higher Motility in Non-Uniform Chemical Landscapes

**DOI:** 10.1371/journal.pone.0015274

**Published:** 2011-04-11

**Authors:** Carl Giuffre, Peter Hinow, Ryan Vogel, Tanvir Ahmed, Roman Stocker, Thomas R. Consi, J. Rudi Strickler

**Affiliations:** 1 Great Lakes WATER Institute, University of Wisconsin - Milwaukee, Milwaukee, Wisconsin, United States of America; 2 Department of Mathematical Sciences, University of Wisconsin - Milwaukee, Wisconsin, United States of America; 3 School of Medicine, Saint Louis University, St. Louis, Missouri, United States of America; 4 Department of Civil and Environmental Engineering, Massachusetts Institute of Technology, Cambridge, Masschusetts, United States of America; University of New Mexico, United States of America

## Abstract

We study the motility behavior of the unicellular protozoan *Paramecium tetraurelia* in a microfluidic device that can be prepared with a landscape of attracting or repelling chemicals. We investigate the spatial distribution of the positions of the individuals at different time points with methods from spatial statistics and Poisson random point fields. This makes quantitative the informal notion of “uniform distribution” (or lack thereof). Our device is characterized by the absence of large systematic biases due to gravitation and fluid flow. It has the potential to be applied to the study of other aquatic chemosensitive organisms as well. This may result in better diagnostic devices for environmental pollutants.

## Introduction

Paramecium is a well-studied genus (*Paramecium*, O. F. Müller, 1773) of unicellular eukaryotic organisms from the class of ciliates that live in freshwater environments [1]. They are shaped like prolate spheroids of 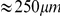 length. The whole body is covered with cilia, with whose help the organisms can swim forward, backward and turn. A sensory apparatus allows to detect temperature, light, and a variety of attracting and repelling chemical substances. The excitable membrane and the predictable behavioral responses make *Paramecium* an appropriate model organism [2].

**Figure 1:**
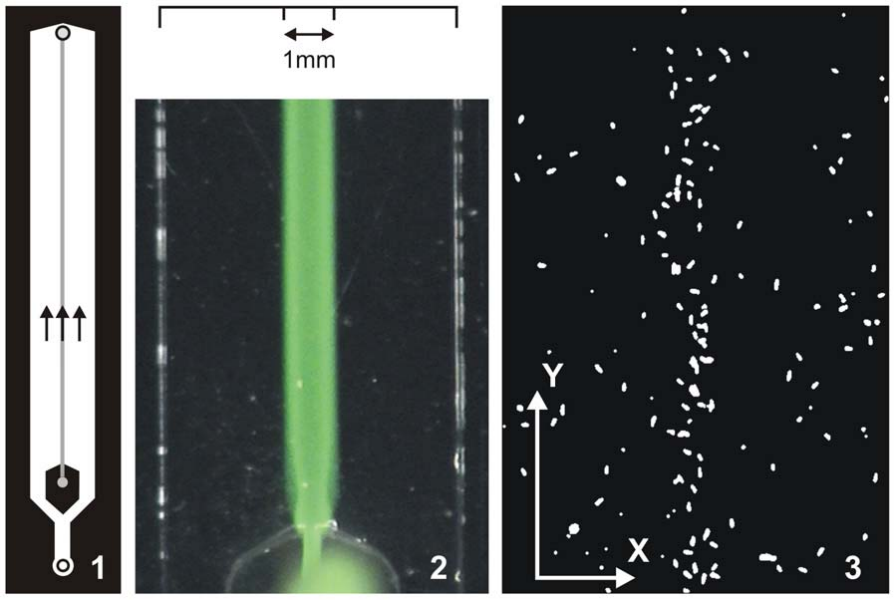
Experimental setup of the microfluidic device. (*Left panel*) Schematic diagram of the microfluidic device. The channel contains two inlets towards the lower end of the channel. One inlet (in the block) serves the middle section while the other inlet delivers fluid for the two side sections. The outlet is the circle on the top end of the channel. (*Middle panel*) Fluorescein was used to visualize the 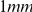 central band, which would contain the test chemical during an experiment. This image shows the central band immediately after the syringe pump was shut off. (*Right panel*) View of *Paramecium* individuals in a small window of the microfluidic device when the center section is loaded with an attracting chemical.

The chemosensitivity of *Paramecium* makes it a potential biosensor for environmental pollutants such as mineral oil, pesticides, urban runoff and others. It is important to understand, in laboratory experiments at first, how *Paramecium* detects its chemical environment and how it translates that information into behavioral changes. Here, we present a novel behavioral assay that targets the chemosensory response of *Paramecium*. Its core is a microfluidic device fabricated with soft lithography using polydimethylsiloxane (PDMS, see Figure 1, left panel). A channel is created with three side-by-side sections of fluids (see Figure 1, middle panel). The dimensions of the device are small enough to neglect turbulent mixing and big enough to neglect molecular diffusion during 2 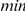 observations. Each section can be loaded with attracting or repelling chemicals and/or a family of approximately 200 individual *Paramecia*. The individuals enter the device at one side either centrally or dispersed over the entire length of that side. The horizontal alignment of the device excludes any systematic bias due to the gravitational field. The motion of the individuals is followed by videomicroscopy under dark field illumination at 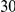 frames per second. The recorded positions in specific frames are then subjected to rigorous statistical analysis.

A device similar to ours was used in recent work by Seymour *et al.* [3], where the authors investigated chemoattraction to dimethylsulfoniopropionate (DMSP) and related compounds in various marine microorganisms. The authors showed a clear chemoattraction in some species by calculating the *chemotactic index* 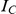, that depends on the ratio between the number of individuals in the domain loaded with the attracting chemical to the number of individuals in the unloaded domains. While such a ratio can be used to demonstrate the chemoattraction, it does not allow more careful analysis and statistical hypothesis testing. The goal of the present paper is to introduce spatial point processes into the study of motility of microorganisms.

Point processes have been studied extensively and have found many applications [4], [5], [6], ranging for example from the distribution of trees in a forest to the distribution of stars and galaxies in the universe. In the remainder of this section we define and give examples for random point processes. We take the unit interval 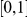 as the underlying state space. Let 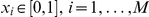 be a finite number of points that we call collectively a *point process* or *point field* 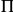. We now review the concept of a spatial Poisson process, first with uniform and then with variable intensity. For background information on the Poisson process we refer to [7].

Let 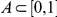 be a *test set* (for simplicity one can think of intervals and their unions) and let 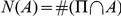 be the number of points of 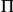 in 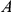. Then the random variables 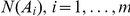 are independent for every family of 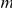 pairwise disjoint sets 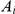. Further, 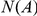 is distributed according to a Poisson distribution with parameter 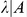, where 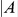 stands for the Lebesgue measure of 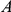 and 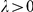 is called the *intensity* of the process. For example, if 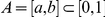 is an interval of length 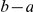, then the probability of finding 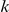 individuals in 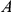 is given by
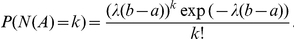
A process where the intensity 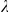 is a constant is called a *homogeneous Poisson process*. More generally, the intensity of the point process can be spatially nonuniform (for example, as in trees in a mountain forest, where the tree density decreases with increasing altitude). Let 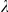 be an integrable, nonnegative function. Then a spatial Poisson process with intensity function 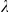 satisfies
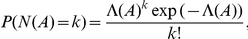
where
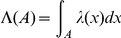
is the expected number of points in the set 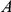. The estimate for the intensity of a uniform Poisson process is 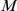, the total number of points (notice that we have normalized the length of the spatial domain to 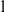). We want to test the null hypothesis that an empirically given point process 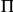 with values in the unit interval 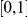 is a uniform Poisson process with intensity 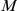. To this end, we divide the interval 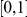 into 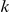 subintervals of equal length 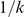 (with 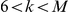) and let 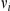 be the number of points of 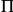 in subinterval 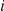. If 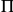 is a uniform Poisson process, then the 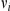 are independent and identically distributed with an average of 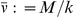 points in each of these subintervals. We calculate the *dispersion index* [4, Chapter 13], [6]

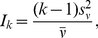
(1)where 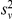 is the sample variance of the point numbers 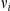. Let 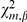 be the 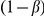-quantile of the 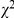-distribution with 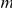 degrees of freedom. Then the hypothesis of a homogeneous Poisson distribution is rejected, if
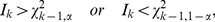
(2)where 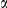 is the probability of an error of type I (rejection of a correct null hypothesis). The smaller 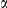 is selected, the wider is the gap between the lower and upper rejection boundaries. In the first rejection case, the points appear to be too much clustered, while the second rejection case, the points appear to be too homogeneous. To improve the confidence in our decision, we calculate the dispersion index for a range of partitions with different numbers of subintervals. The larger the number of points 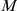, the finer are the contrasts (i.e. the deviations from a homogeneous Poisson distribution) that can be detected by the above rejection method.

## Results

The microfluidic device consists of three parallel sections aligned in the direction of the 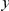-axis, see Figure 1. Two point processes are obtained by extracting the positions of individual *Paramecium* on certain frames, we denote these by 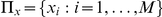 and 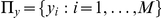, respectively. These two processes are normalized so that they both take values in 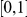.

**Figure 2:**
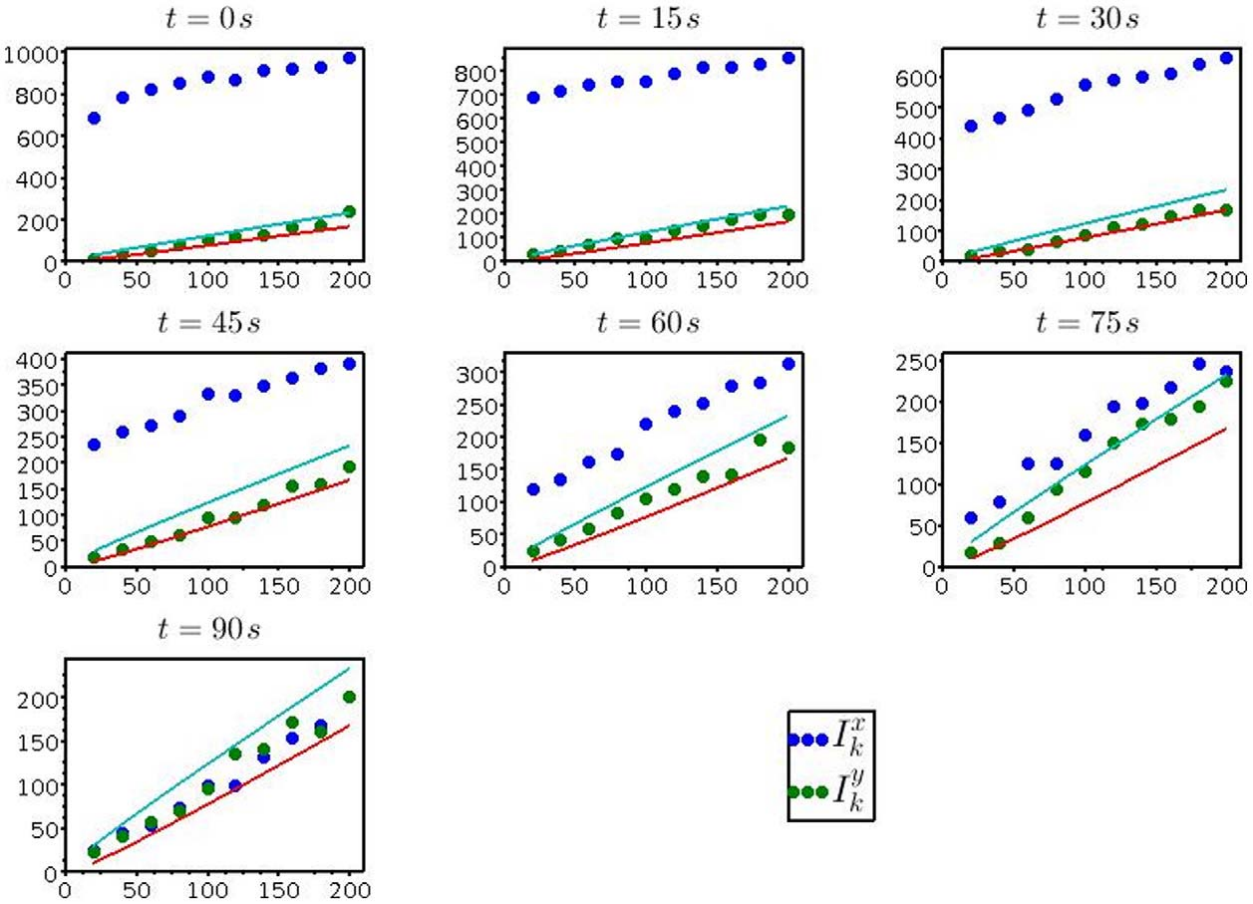
Dispersion indices of the point processes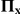 and 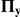 at different times of the video, where the central section is not loaded with any chemical. The solid lines are the lower and upper rejection bounds from equation (2) with error probability 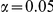. Data points above the upper rejection bound indicate that the point process is too much clustered to be a homogeneous Poisson process. The dispersion index in the 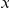-direction approaches that of a homogeneous Poisson process over a time of 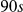 while the dispersion index in the 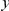-direction is that of a homogeneous Poisson process throughout.

The first video of total duration of 2 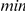 was taken as a control in a microfluidic device not prepared with either attracting or repelling chemicals. The individuals enter the device in the middle third of the interval 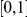 in the 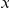-direction. We calculate the dispersion indices from equation (1) to test the hypothesis of a homogeneous Poisson process, for both the processes 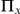 and 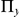. The number of individuals in every frame is approximately 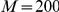. The results are shown in Figure 2. We see that the point process 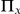 becomes more and more homogeneous over the duration of the experiment, while 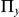 is homogeneous at all times.

**Figure 3:**
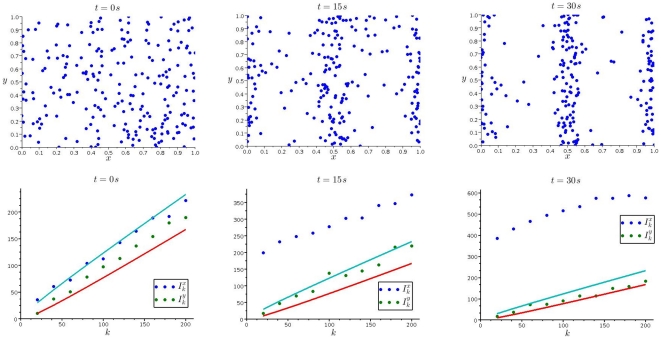
Aggregation of *Paramecium* subjected to an attractant. (*Top row*) Positions of 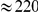 *Paramecium* individuals after 0, 15 and 30 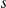 (from left to right), when the center section is loaded with 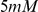 of the attractant sodium acetate. (*Bottom row*) The corresponding dispersion indices in 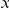
- (blue) and 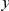-directions (red).

In the second video, the individuals are injected over the whole width of the 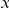-axis and the center section is loaded with 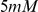 of the attracting substance sodium acetate [8], [9], [10], [11], see Figure 3. Here we see that an initially homogeneous Poisson process 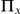 evolves to a three-peaked distribution within 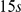. The peaks at 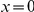 and 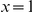 are due to effects of the walls on the *Paramecium*. It has been established that forces from the walls exert drag on the microorganisms, due to their movement at such low Reynolds numbers [12]. This phenomenon may be of occasional nature. The dispersion index of the process 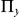 shows no significant deviation from a homogeneous Poisson process in the direction of the three sections (the 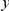-axis) at any time.

**Figure 4:**
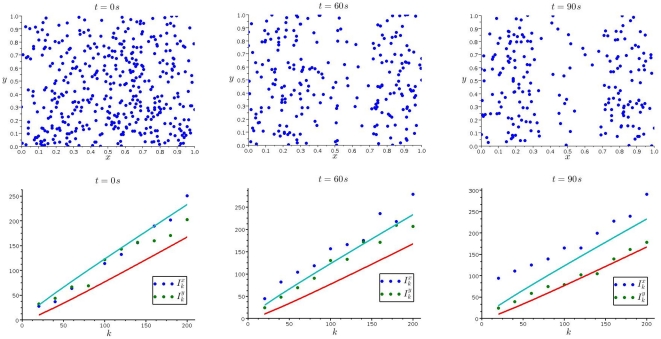
Dispersion of *Paramecium* subjected to a repellent. (*Top row*) Positions of 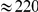 *Paramecium* individuals after 0, 60 and 90 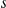 (from left to right), when the center section is loaded with 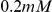 of the repellent potassium ferricyanide. (*Bottom row*) The corresponding dispersion indices in 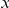
- (blue) and 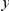-directions (red).

In the third video, the individuals are again injected over the whole width of the 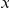-axis and the center section is loaded with 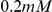 of the repelling substance potassium ferricyanide [13], see Figure 4. Interestingly, emptying the center strip takes longer than accumulation in the center strip if it is loaded with an attractant.

## Discussion

Spatial statistics and random point fields have been successfully applied in many situations, an important source of inspiration being ecological questions [4], [5], [6]. As examples we mention the distributions of trees in a forest, nests and burrows in a habitat or the spread of diseases by contact across large distances. Here we apply Poisson point processes to the motion of *Paramecium tetraurelia* in a microfluidic device with possible attracting or repelling substances. While a pattern is clearly recognizable from the raw point plots in the top row of Figure 3, the statistical rejection method has the advantage that it is quantitative and reproducible. Moreover, the fact that the distribution in 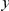-direction should not, and indeed *does not* change, serves as a control to rule out undue disturbances from the fluid flowing through the device.

Motile organisms and cells sense their environment and react to it by directed motion, a process that is usually called *taxis*. This behavior has been studied widely both at the experimental and theoretical level, see [14],[15],[16] for groundbreaking early works and [17], [18] for some recent contributions. When studying the motion of cells or organisms, one has to distinguish between a directed motion towards (or away from) a source and a counteracting random motility that can be compared to Brownian motion of suspended particles in a heat bath as it was studied by Albert Einstein [19]. These two opposing behaviors enter the so-called Keller-Segel model of chemotaxis, of which the equation for the motile individuals reads
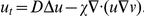
Here 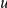 is the population density of the moving species, while 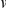 is the density of the chemical substance that provides the cue for the taxis. The constant 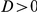 is the equivalent of the Fickian diffusion coefficient. The gradient 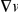 gives the direction of the chemosensory motion and the chemotactic sensitivity 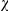 is 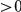 for an attracting and 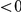 for a repelling substance. The main result of the present paper is that a motion towards an attracting source occurs faster (Figure 3) than the dispersion of the individuals in a flat chemical landscape that would be attributed to random motion alone (Figure 2). A precise determination of the constant 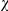 requires the control of the gradients of attracting or repelling substances. This will be addressed in future work.

Our device and our method of data analysis can be applied to a variety of aquatic microorganisms and attracting or repelling chemicals. Similarly, in testing different compounds at different concentrations, Seymour *et al.* [3] showed that their organisms reacted species specifically. The question then is whether the speed is correlated with the strength of the dissolved chemical compound, the concentration and/or its efficacy, or whether it is a diffusion problem considering the boundaries of the microfluidic devices, the behaviors of the different organisms, and the different chemical landscapes across the experiments. The result that the motion to and fro a chemical source occurred at different speeds will generate further research.

## Materials and Methods

### Cell cultures

*Paramecium tetraurelia* type 51s was obtained as a gift from Dr. Thomas G. Doak, (University of Indiana). The *Paramecium* cells were grown at 24 
C in monoxenic cultures consisting of a sterile complex protozoan medium (Carolina Biological Supply Company, Burlington, NC) inoculated with *Klebsiella pneumonia*.

### Cell preparation

The cells were harvested at early stationary growth phase (4–7 days) and concentrated. We concentrated the *Paramecium* cells by passing the liquid culture medium through nylon mesh membranes (Small Parts, Inc., Miramar, FL). Membranes with 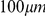 and 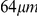 pores were used first to remove debris. A membrane with 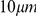 pores was then utilized, which stopped the *Paramecium* cells but allowed liquid and bacteria to pass through. The cells were then washed by replacing the growth medium liquid with resting buffer solution using a nylon membrane with 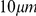 pores. The resting buffer solution consisted of (mM): 4 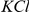, 1 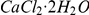, and 1 tris- 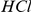 (pH 7.0).

### Microfluidic device

The microfluidic device was fabricated with PDMS using soft lithography and was mounted on a glass slide as described in [20]. It contained a channel that was 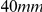 long, 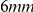 wide, and 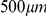 deep. The channel had one inlet for the middle section, one inlet for the two side sections, and an outlet at the opposite end (Figure 1, left panel). When a test chemical entered the channel, it created a coherent central band, which we visualized with fluorescein (Figure 1, middle panel).

### Experimental conditions

Washed and concentrated *Paramecium* cells (approximately 12,000 cells/ 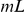) along with a possible test chemical were injected into the sections simultaneously through the two separate inlets via two syringes (1000 series; Hamilton) and an actuator (model # 850-2; Newport Corporation). When the actuator was activated, fluid was delivered from the syringes at a ratio of 5∶1, creating a 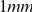 central band containing a test chemical, surrounded by two lateral bands containing *Paramecium* cells (Figure 1, right panel). The actuator created a flow of 3 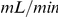 through the channel, which was rapid enough so that the width of the central band was essentially the same throughout the length of the channel. When the actuator was shut off, flow stopped immediately and the test chemical gradually diffused laterally in the channel. The channel was rinsed with 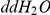 after each run. All experiments were done at a temperature of 24 
C.

### Known attractants and repellents

We tested known attractants and repellents on *Paramecium* to determine the efficacy of the microfluidic channel for observing chemoresponse behavior of this organism. The known attractant that we used was sodium acetate 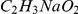 [8], [9], [10], [11]. We filled the central band syringe with our resting buffer, along with 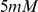 of sodium acetate as the test chemical. The lateral band syringe consisted of *Paramecium* cells in our resting buffer, along with 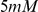 of 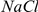 to balance the osmolarity of the central band. The known repellent that we used in our experiment was potassium ferricyanide 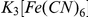 [13]. The central band syringe in this repellent experiment was loaded with our resting buffer along with 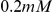 of potassium ferricyanide as the test chemical, and the lateral band syringe consisted of *Paramecium* cells in our resting buffer along with an additional 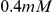 of 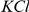 to balance the osmolarity of the central band. We also tested controls in which no test chemical was added to the central band.

### Data acquisition and analysis

The *Paramecium* cells were imaged with a camera (XC-EI50; Sony) connected to a stereo microscope (Zeiss) under near-infrared dark field illumination at 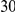 frames per second. The images were analyzed with ImageJ (NIH; Bethesda, MD). The 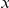
- and 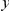-positions of individuals were stored in ASCII text files. The analysis software was written with the open source package scilab [21]. The raw data, the position files and the analysis software are available as supporting information S1.

## Supporting Information

Supporting Information S1 **The suppotring information contains the raw positional data of the** ***Paramecium*** **individuals and the scilab software that is used to analyze them.**
(ZIP)
