# Supplementary figures and images for: The Ciliate Paramecium Shows Higher Motility in Non-Uniform Chemical Landscapes
Source: PLoS One. 2011 Apr 11;6(4):e15274. doi: 10.1371/journal.pone.0015274 (PMC3073933; doi:10.1371/journal.pone.0015274)

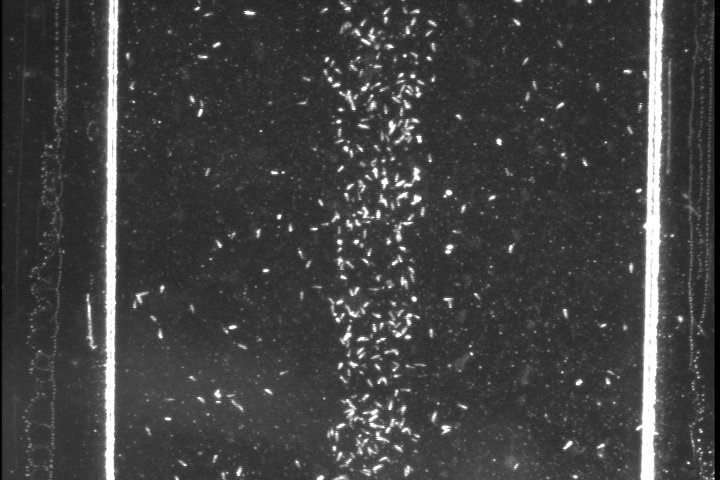

Supplement: Supporting Information S1 — The suppotring information contains the raw positional data of the Paramecium individuals and the scilab software that is used to analyze them. (ZIP) [file pone.0015274.s001.zip › ControlAt0.tif]

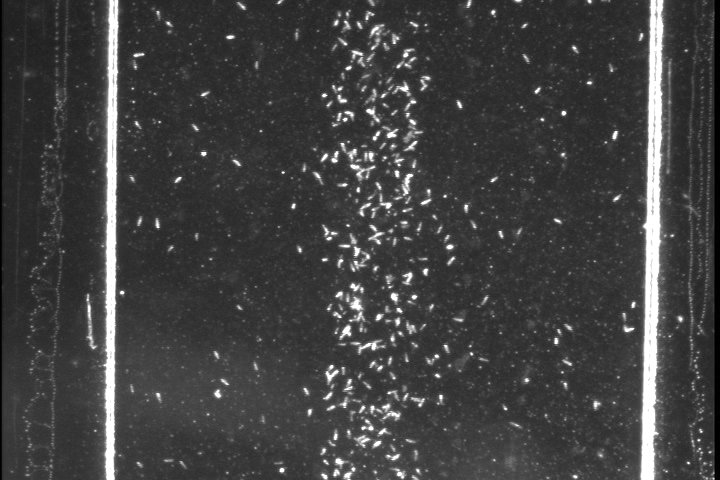

Supplement: Supporting Information S1 — The suppotring information contains the raw positional data of the Paramecium individuals and the scilab software that is used to analyze them. (ZIP) [file pone.0015274.s001.zip › ControlAt15.tif]

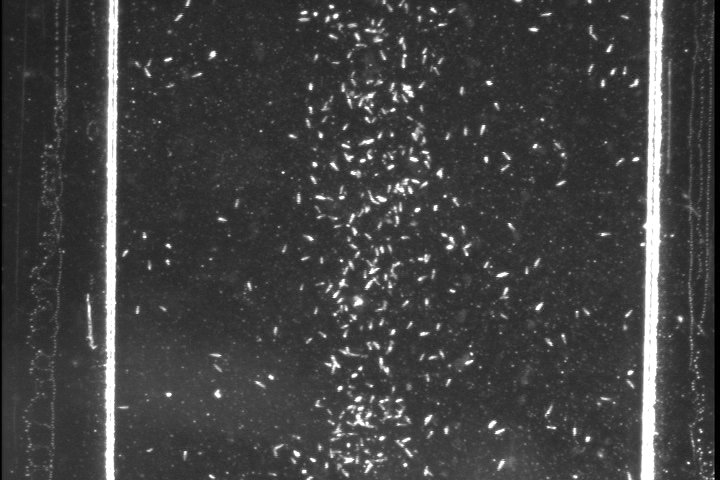

Supplement: Supporting Information S1 — The suppotring information contains the raw positional data of the Paramecium individuals and the scilab software that is used to analyze them. (ZIP) [file pone.0015274.s001.zip › ControlAt30.tif]

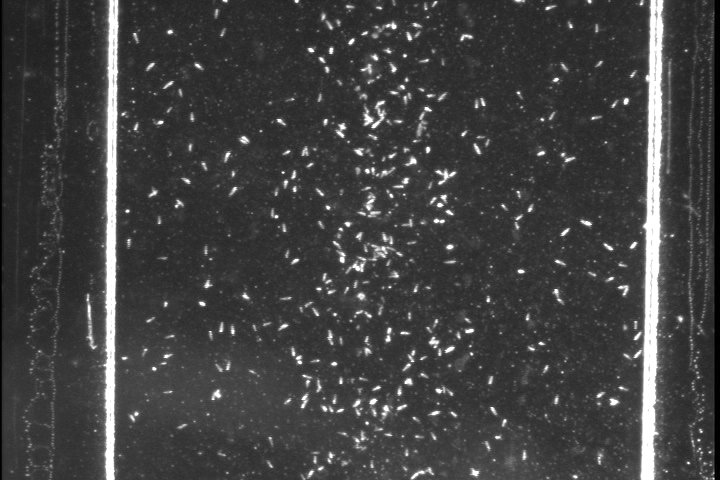

Supplement: Supporting Information S1 — The suppotring information contains the raw positional data of the Paramecium individuals and the scilab software that is used to analyze them. (ZIP) [file pone.0015274.s001.zip › ControlAt45.tif]

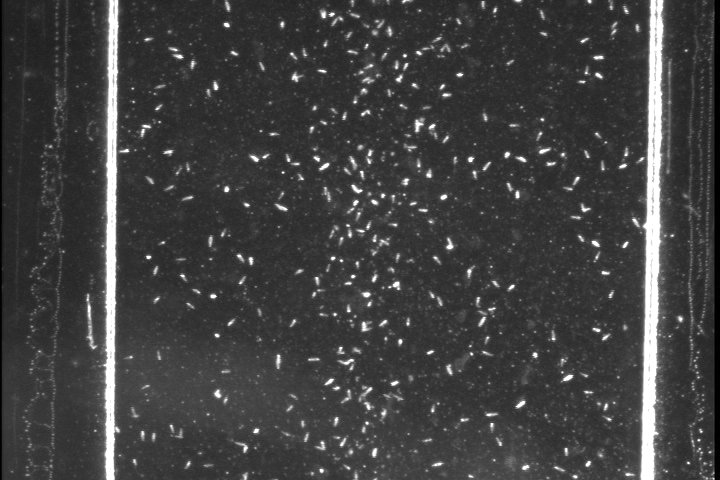

Supplement: Supporting Information S1 — The suppotring information contains the raw positional data of the Paramecium individuals and the scilab software that is used to analyze them. (ZIP) [file pone.0015274.s001.zip › ControlAt60.tif]

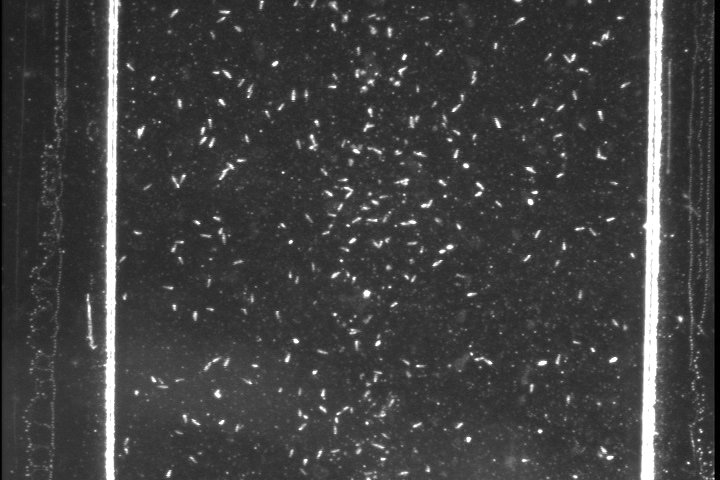

Supplement: Supporting Information S1 — The suppotring information contains the raw positional data of the Paramecium individuals and the scilab software that is used to analyze them. (ZIP) [file pone.0015274.s001.zip › ControlAt75.tif]

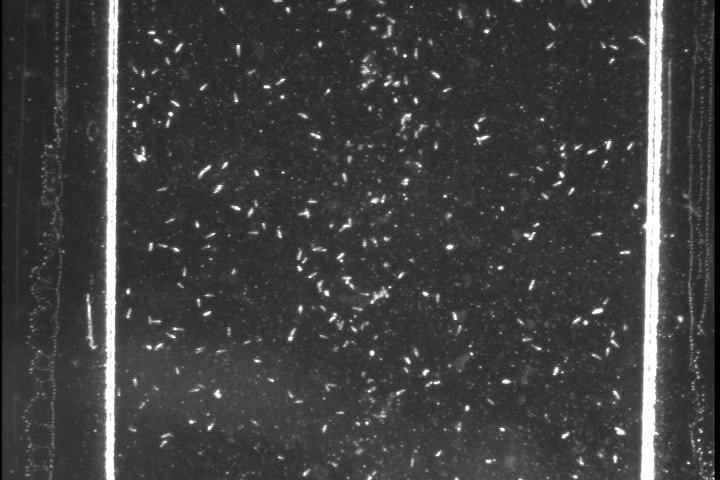

Supplement: Supporting Information S1 — The suppotring information contains the raw positional data of the Paramecium individuals and the scilab software that is used to analyze them. (ZIP) [file pone.0015274.s001.zip › ControlAt90.tif]

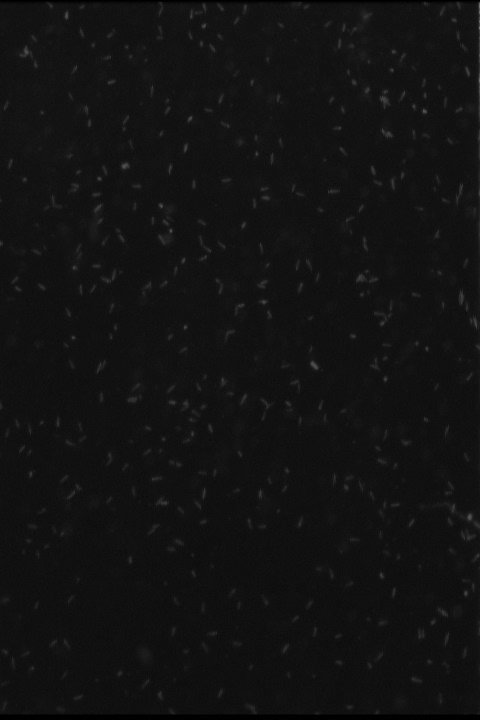

Supplement: Supporting Information S1 — The suppotring information contains the raw positional data of the Paramecium individuals and the scilab software that is used to analyze them. (ZIP) [file pone.0015274.s001.zip › RepellentAt0.tif]

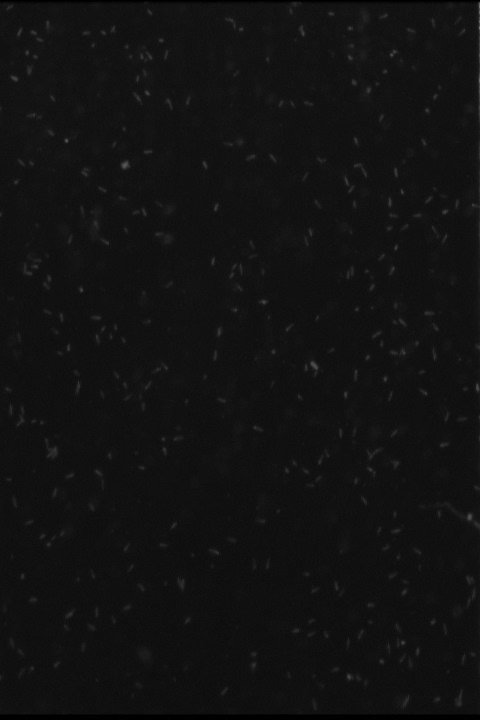

Supplement: Supporting Information S1 — The suppotring information contains the raw positional data of the Paramecium individuals and the scilab software that is used to analyze them. (ZIP) [file pone.0015274.s001.zip › RepellentAt30.tif]

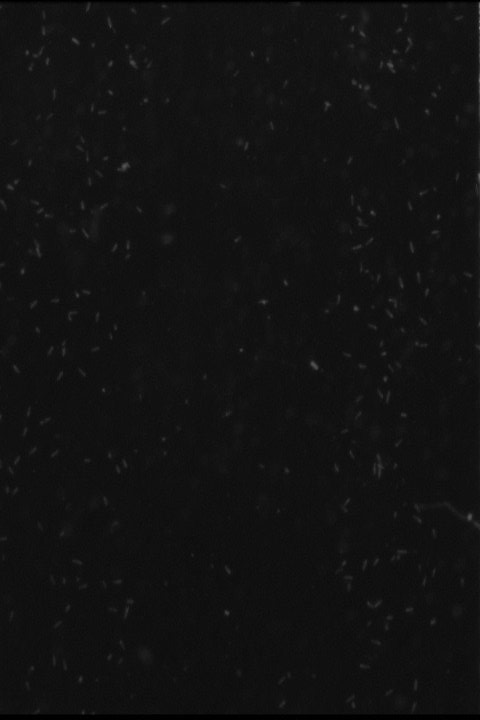

Supplement: Supporting Information S1 — The suppotring information contains the raw positional data of the Paramecium individuals and the scilab software that is used to analyze them. (ZIP) [file pone.0015274.s001.zip › RepellentAt90.tif]

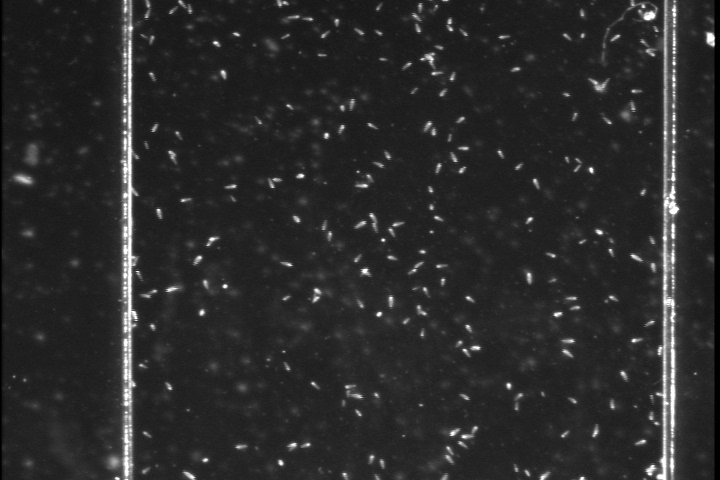

Supplement: Supporting Information S1 — The suppotring information contains the raw positional data of the Paramecium individuals and the scilab software that is used to analyze them. (ZIP) [file pone.0015274.s001.zip › AttractantAt0.tif]

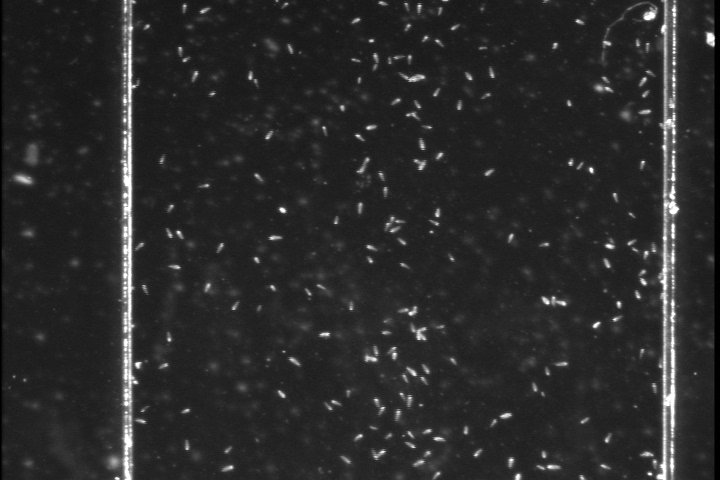

Supplement: Supporting Information S1 — The suppotring information contains the raw positional data of the Paramecium individuals and the scilab software that is used to analyze them. (ZIP) [file pone.0015274.s001.zip › AttractantAt5.tif]

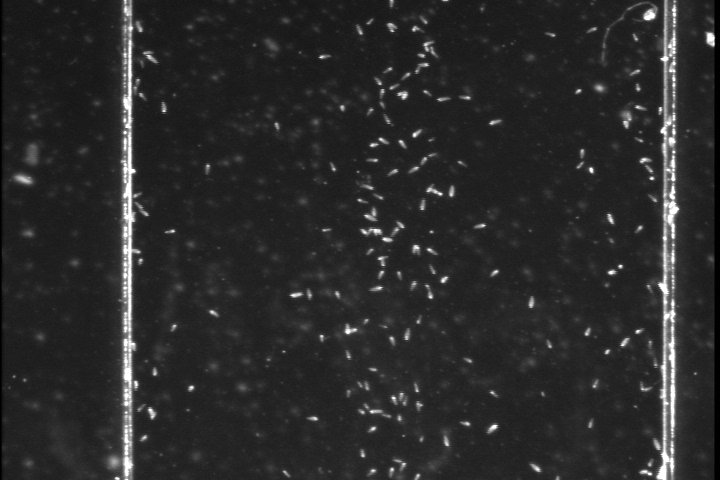

Supplement: Supporting Information S1 — The suppotring information contains the raw positional data of the Paramecium individuals and the scilab software that is used to analyze them. (ZIP) [file pone.0015274.s001.zip › AttractantAt15.tif]

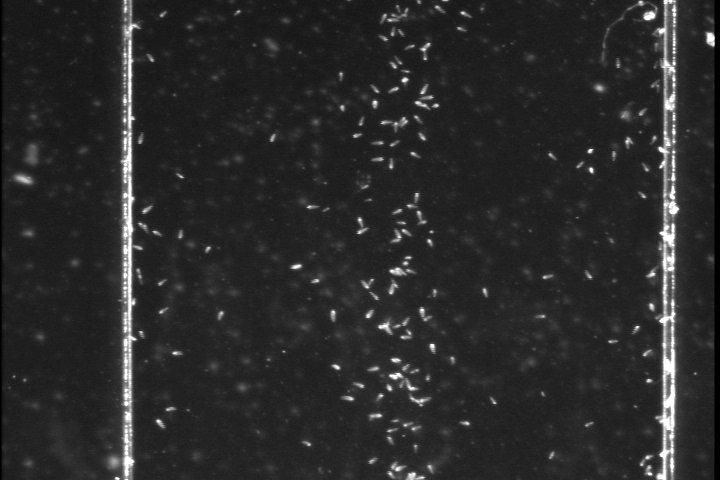

Supplement: Supporting Information S1 — The suppotring information contains the raw positional data of the Paramecium individuals and the scilab software that is used to analyze them. (ZIP) [file pone.0015274.s001.zip › AttractantAt30.tif]

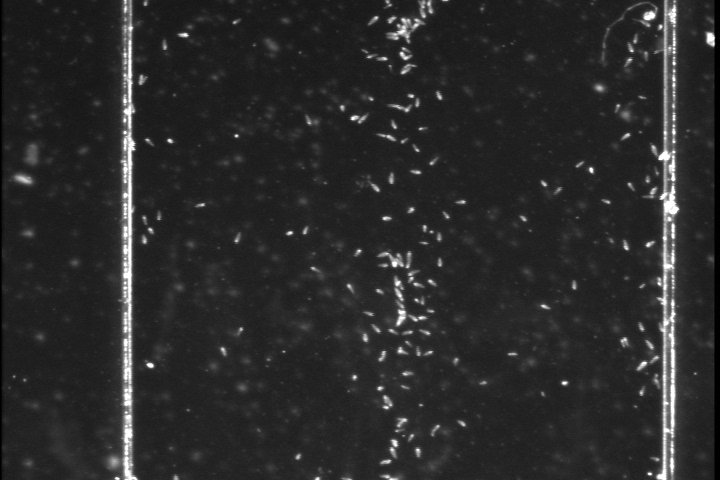

Supplement: Supporting Information S1 — The suppotring information contains the raw positional data of the Paramecium individuals and the scilab software that is used to analyze them. (ZIP) [file pone.0015274.s001.zip › AttractantAt60.tif]
